# Dried Whole Plant *Artemisia annua* as an Antimalarial Therapy

**DOI:** 10.1371/journal.pone.0052746

**Published:** 2012-12-20

**Authors:** Mostafa A. Elfawal, Melissa J. Towler, Nicholas G. Reich, Douglas Golenbock, Pamela J. Weathers, Stephen M. Rich

**Affiliations:** 1 Laboratory of Medical Zoology, Department of Microbiology, University of Massachusetts, Amherst, Massachusetts, United States of America; 2 Department of Biology and Biotechnology, Worcester Polytechnic Institute, Worcester, Massachusetts, United States of America; 3 Division of Biostatistics and Epidemiology, School of Public Health and Health Sciences, University of Massachusetts, Amherst, Massachusetts, United States of America; 4 Infectious Diseases and Immunology, University of Massachusetts Medical School, Worcester, Massachusetts, United States of America; Université Pierre et Marie Curie, France

## Abstract

Drugs are primary weapons for reducing malaria in human populations. However emergence of resistant parasites has repeatedly curtailed the lifespan of each drug that is developed and deployed. Currently the most effective anti-malarial is artemisinin, which is extracted from the leaves of *Artemisia annua*. Due to poor pharmacokinetic properties and prudent efforts to curtail resistance to monotherapies, artemisinin is prescribed only in combination with other anti-malarials composing an Artemisinin Combination Therapy (ACT). Low yield in the plant, and the added cost of secondary anti-malarials in the ACT, make artemisinin costly for the developing world. As an alternative, we compared the efficacy of oral delivery of the dried leaves of whole plant (WP) *A. annua* to a comparable dose of pure artemisinin in a rodent malaria model (*Plasmodium chabaudi*). We found that a single dose of WP (containing 24 mg/kg artemisinin) reduces parasitemia more effectively than a comparable dose of purified drug. This increased efficacy may result from a documented 40-fold increase in the bioavailability of artemisinin in the blood of mice fed the whole plant, in comparison to those administered synthetic drug. Synergistic benefits may derive from the presence of other anti-malarial compounds in *A. annua*. If shown to be clinically efficacious, well-tolerated, and compatible with the public health imperative of forestalling evolution of drug resistance, inexpensive, locally grown and processed *A. annua* might prove to be an effective addition to the global effort to reduce malaria morbidity and mortality.

## Introduction

Malaria is among the most prevalent infectious diseases in the developing world, imposing a vast burden of mortality and perpetuating cycles of poverty. In 2009, the World Health Organization (WHO) estimated that 225 million cases of malaria occurred, with >780,000 deaths [1]. In spite of recent advances in our understanding of this parasite, efforts to prevent transmission have remained largely unchanged for over a century. Though malaria vaccines hold future promise, vector control and chemotherapy remain the primary weapons for reducing the burden of disease in individuals and populations. Artemisinin, in the form of Artemisinin-based Combination Therapy (ACT), is currently the best treatment option against those malaria parasites that have evolved resistance to drugs such as chloroquine [2]. Moreover, artemisinin (AN) and its derivatives have also been shown to affect a number of viruses, a variety of human cancer cell lines [3], [4], several neglected tropical parasitic diseases including schistosomiasis [5], leishmaniasis [6], [7], New- and Old-World trypanosomiases [8], and some livestock diseases [9].

Current artemisinin production requires its extraction from the cultivated herb *Artemisia annua* L., which is a “generally regarded as safe” (GRAS) herb suitable for human consumption [10]. However, considerable production costs and inadequate availability of artemisinin limit its present usefulness in the campaign against malaria [11]. Until very recently [12], *de novo* chemical synthesis of artemisinin was neither practical nor cost-effective. The best plant cultivars yield only *ca*. 1.5% artemisinin, and agricultural yields seldom exceed 70 kg/ha [13]. The drug is solvent-extracted from plant material, crystallized, and typically used for semi-synthesis of artemisinic derivatives. Although *A. annua* is relatively easy to grow in temperate and subtropical climates, low yields of artemisinin lead to relatively high costs for isolation and purification of the drug [14]. Because of this shortcoming, plant scientists have focused their efforts on producing cultivars of *A. annua* with higher artemisinin crop yield [15]. Transgenic production schemes are also underway [16], [17]. Meanwhile, a worldwide shortage fails to meet the need to treat malaria, not to mention those other diseases against which artemisinin holds such promise [2].

One means of reducing the cost of production would be to limit the amount of post-harvest processing by using the whole plant (WP). We wondered whether omitting the extraction step, by using whole plant *A. annua* directly as the source of artemisinin, might prove efficacious in an experimental murine model. In a previous study, we showed that mice fed dried WP material had about 40 times more artemisinin in their bloodstream than mice that were fed a corresponding amount of pure drug [10]. This amount exceeded by eight fold the minimum concentration of serum artemisinin (10 µg/L) required against *P. falciparum* [18]. This suggested that the active ingredients were delivered faster, and in greater quantity, from whole plant treatments than from pure drug treatments. We further hypothesized that because of the combination of parasite-killing substances normally present in the plant (artemisinin and flavonoids) [19], [20], a synergism among these constituent compounds might render whole plant consumption as a form of ACT.

We thus sought to determine whether WP *A. annua* can kill malaria parasites *in vivo* using a rodent malaria model. *Plasmodium chabaudi* is an excellent model for the most deadly of the human parasites, *P. falciparum*, because both species demonstrate preference for mature erythrocytes as opposed to reticulocytes that are favored by other human and rodent malaria parasites [21], [22]. Rodent malaria models are invaluable for studying modes and mechanisms of resistance to antimalarial compounds [23]. Demonstrating parasite killing using this inexpensive and resilient treatment could be an important first step in establishing a novel therapy based on WP *A. annua* for treatment of human malaria. Furthermore, it may help to identify other complementary and/or synergistic anti-malarial compounds within the plant.

## Results and Discussion

**Figure 1:**
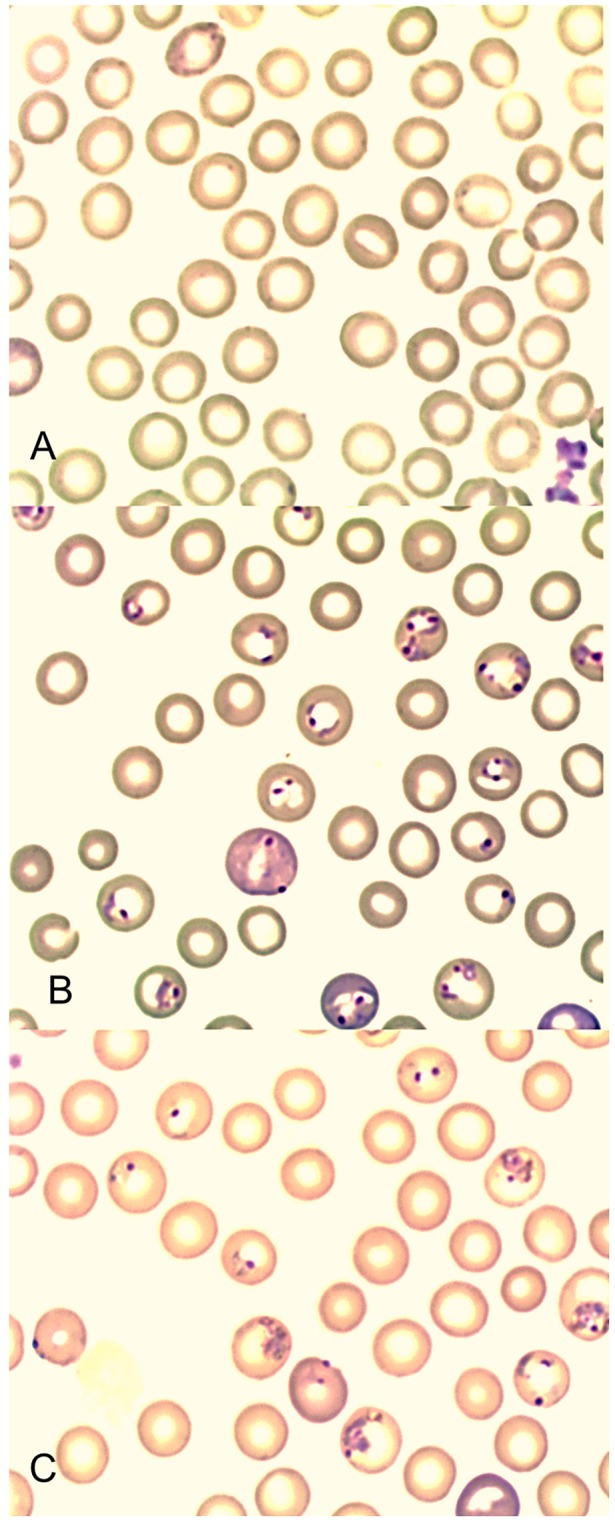
Giemsa stained blood smears from mice showing erythrocytes infected with *P. chabaudi*, 30 **hours after treatment with** (**A**) **WP^LO^** (**24** **mg/kg whole plant delivered artemisinin**)**,** (**B**) **AN^LO^** (**24** **mg/kg pure drug artemisinin**)**, and** (**C**) **Placebo control.**

**Figure 2:**
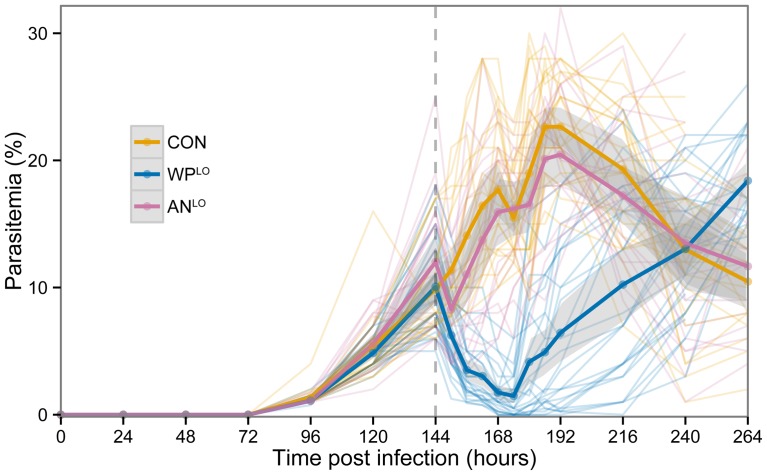
*P. chabaudi* parasitemia of three treatment groups across all experimental replicates: low-dose whole plant *A. annua* (**WP^LO^, N = 26 containing 24 mg/kg** ***in planta*** **artemisinin, and low-dose pure drug** (**AN^LO^, N = 20**) **containing 24 mg/kg pure artemisinin, and placebo control** (**CON, N = 23**) **which received only mouse chow.** Dark lines indicate average parasitemia by treatment type. Lighter lines show individual mouse trajectories. Shaded regions indicate the 95% confidence interval for the means calculated based on normality assumptions. Dashed vertical line indicates time of treatment.

We found conclusive evidence that orally ingested, powdered dried leaves of whole plant *A. annua* kills malaria parasites more effectively than a comparable dose of pure drug. In our primary analysis we used dried *A. annua* leaves containing 14.8 mg artemisinin per gram of dried leaves and compared parasitemia over time in mice treated with either low-dose, whole plant *A. annua* (WP^LO^), low-dose, pure drug artemisinin (AN^LO^), or placebo (CON). Only 24 hr after treatment, dead parasites (with condensed dark pigment) were observed in mice treated with WP^LO^; 30 hours after treatment, parasitemia was <0.001% (Figure 1). Mice treated with WP^LO^ showed significantly lower parasitemia than those treated with AN^LO^ from 12 to 72 hours post-treatment (Figure 2). Mice treated with AN^LO^ did not show significant difference in parasitemia from those administered a placebo at any time point.

**Figure 3:**
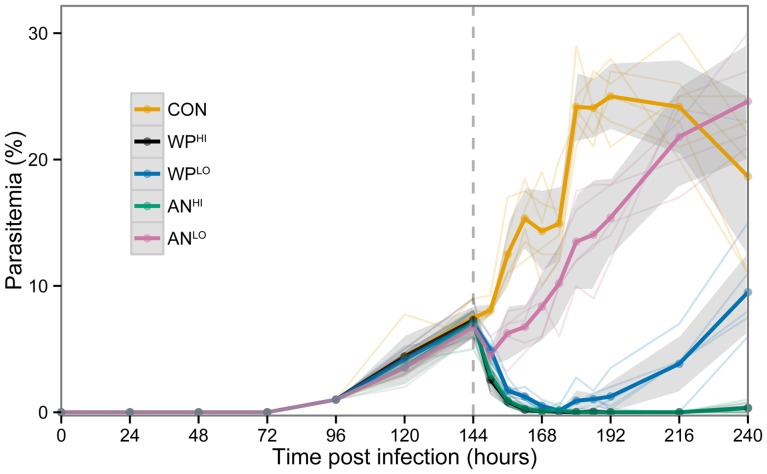
Dose comparisons of WP and AN treatments from the third replicate of data: WP^LO^ (**N = 6**) **and WP^HI^** (**N = 6**) **received 24 mg/kg and 120 mg/kg** ***in planta*** **artemisinin, respectively; AN^LO^** (**N = 5**) **and AN^HI^** (**N = 6**) **received 24 mg/kg and 120 mg/kg pure artemisinin, respectively.** Placebo control (CON, N = 6) received only mouse chow. Dark lines indicate average parasitemia by treatment type. Lighter lines show individual mouse trajectories. Shaded regions indicate the 95% confidence interval for the means calculated based on normality assumptions. Dashed vertical line indicates time of treatment.

In a subsequent dose-response analysis, we added high-dose comparison groups, AN^HI^ and WP^HI^. Each of the four treatment groups experienced significantly lower parasitemia than the control mice (Figure 3). All mice treated with WP^LO^ responded strongly in the first 24 hr following gavage, showing the lowest level of parasitemia at 30 hours post-gavage; only 3 of 26 WP^LO^-treated mice (12%) had parasitemia ≥3%. However, 36 hours after gavage, 38% of the WP^LO^-treated mice were above this threshold. Notably, treatment with WP^LO^ was just as effective in reducing parasitemia as was treatment with AN^HI^ for the first 72 hours post treatment (Figure 3). Thereafter, WP^LO^-treated mice had significantly higher parasitemia. Although the suppression of parasitemia was significant in the three treatment groups after a single dose treatment, low dose WP^LO^ resulted in faster recrudescence than either WP^HI^ or AN^HI^ (Figure 3), suggesting that multiple treatments at this dose would be necessary for a curative effect. Considering that a normal course of ACT treatment for human malaria currently requires several doses, spread over several days, a similar regime may also be effective in the case of lower dose WP administration. Pharmacokinetics will be needed to determine serum levels of the drug. No differences between the WP^HI^ and AN^HI^ groups were detected after treatment. This is consistent with the short half-life seen in the pure drug in human patients and the prescribed need for multiple doses per day for several days [24].

Although the precise mechanism of its anti-malarial activity remain unproven, artemisinin is suspected (like other drugs, including chloroquine) to interfere with heme detoxification, a crucial requirement for parasite survival in erythrocytes. *Plasmodium* parasites digest hemoglobin, producing heme as a byproduct. Free heme molecules are toxic, so parasites sequester it in the form of hemozoin polymers in unique digestive vacuoles. Artemisinin is a sesquiterpene lactone with a crucial endoperoxide bridge [25], and in the presence of heme, this bridge is broken, thereby releasing free radicals harmful to the parasite [26].

The empirical evidence for increased anti-malarial activity of WP relative to AN might be explained by differential bioavailability of the artemisinic compounds. Weathers et al. showed that mice treated with a WP equivalent of 1.2 mg/kg AN reached their highest concentration of artemisinin in the blood (87 µg/L) 30 min after gavage, whereas mice treated with AN did not reach their the maximum concentration (74 µg/L) until much later (≥60 min) [10]. Moreover, poor solubility and high metabolic breakdown of artemisinin by hepatic and intestinal cytochrome P enzymes (CYP P450 and CYP3A4) may reduce its bioavailability when administered in pure form [27]. Infusions made from whole plant *A. annua* showed marked inhibition of the intestinal and hepatic CYP enzymes by flavonoids and/or other compounds [9]. Hence, inhibition of metabolic enzymes correlates with greater bioavailability of artemisinin, which is consistent with our findings that WP demonstrates greater parasite killing activity than a comparable pure drug treatment.

Whole plant (WP) *A. annua* may also have enhanced antimalarial activity due to synergism among particular plant compounds and artemisinin [9], [28], [29]. Among these compounds are many flavonoids, of which at least six (artemetin, casticin, chrysosplenetin, chrysosplenol-D, cirsilineol, and eupatorin) are of interest for their antimalarial roles. The synergism between artemisinin and these flavonoids may be due to their ability to potentiate the activity of artemisinin. When each of these six flavonoids was combined individually with artemisinin, the IC_50_ of AN against *P. falciparum* decreased by 20–50%, demonstrating an apparent synergy between the sesquiterpene lactone, artemisinin, and those six methoxylated flavonoids [19], [20]. The precise mechanism of flavonoids in activating artemisinin is not fully understood, however it has been reported that *A. annua* methoxylated flavonoids enhance the formation of the artemisinin-heme complex [30], which increases the release of free radicals.

Two other major *A. annua* flavonoids, myricetin and quercetin, are known to inhibit mammalian thioredoxin reductase, which is critical for cellular redox control [31]. Thioredoxin reductase is also essential for the *P. falciparum* erythrocytic stage [32]; therefore, inhibition of this parasite enzyme by myricetin and quercetin may work in synergy with artemisinin against *P. falciparum* [33].

In addition to the bioavailability and potentiation attributes of WP, there are other compounds in *A. annua* that may act to reduce parasitemia independent of artemisinin. Liu et al (1992) reported the antimalarial activity of several *A. annua* flavonoids delivered *in vitro* as isolated compounds, in the absence of artemisinin [20]. Moreover, antimalarial activity has been documented for related plant species that do not produce artemisinin [34]. Among the compounds in *A. annua* not yet fully investigated are more than a dozen other sesquiterpenes, some of which have shown promise for killing parasites in rodent models [28].

Determining the mechanisms for increased efficacy of WP will require further investigation, but it seems certain that the constituent compounds contained within *A. annua* comprise a complex set of interactions and synergies yet to be described. Given the complex nature of the plant and its many components, WP may not necessarily be considered a simple monotherapy. While the temptation might be to consider WP as merely an alternative delivery mechanism for artemisinin, our results strongly indicate that WP is unique and may represent an innovative combination therapy. We refer to this as a *plant Artemisinin Combination Therapy* (pACT). A pACT can be distinguished from other combinational therapies where the drug components do not necessarily work synergistically because their combinations are artificially contrived. The pACT comprises a biologically complex entity, in which the combinations are result of evolutionary processes that would have attributes of redundancy and resiliency that make combination therapies selectively advantageous to simple monotherapies. Refinements of these combinations by evolutionary processes ensures they are robust.

The novelty of the WP pACT cannot be overemphasized as there is a common misconception that this therapy has been tested previously. It has not. The WP therapy tested in the present study should not be confused with tea or infusion therapy. Whole plant *A. annua* (WP) tested here against murine malaria, uses the plant leaves, dried under controlled conditions and ingested by the host. Such a preparation of *A. annua* has never been tested against malaria parasites (in humans, mice or otherwise).

Because *A. annua* has long been used to make tea to treat fever in Asia [35], several investigators have proposed to re-establish the use of *A. annua* tea for rapid treatment of malaria [2], [36]. These teas have major shortcomings. First, large volumes of tea must be consumed over short periods to ensure adequate ingestion of drug, a nontrivial matter considering the bitter taste of the tea, especially for pediatric patients. Moreover, while a 5 min boiling water extraction yields about 90% of the plant's artemisinin [36], this is not an effective process for extracting key flavonoids [20]. Our analysis of hot water tea extracts following the optimized protocol described by van der Kooy and Verpoorte [36], showed loss of about 99% of some of the original flavonoids that reportedly synergize with artemisinin [37].

Not only does WP differ from teas and infusions in terms of its efficacy and pharmaceutical properties, but also because of its preparation, it can be carefully controlled and preserved. We previously proposed development of a new form of anti-malarial therapy based on dried, encapsulated *A. annua* leaves as an inexpensive, dose-controlled, rapid delivery of the drug to treat uncomplicated cases of malaria and other neglected tropical diseases for which artemisinin has been shown to be effective [10]. Dosage can be controlled because dried WP *A. annua* can be homogenized and assayed for artemisinin content prior to encapsulation. Capsule size and number can be adjusted based on artemisinin content and patient weight.

Our purpose in the present study was to determine whether this “generally regarded as safe” (GRAS) herb [38] is effective in killing malaria parasites *in vivo*. Extrapolating appropriate human dosage from experimental evidence in mouse models will require additional investigation, however it is generally accepted that this extrapolation does not scale linearly with respect to body mass. Better indicators that allow for allometry include use of total body surface area [39] and/or take full consideration of physiochemical properties of the drugs and species involved [40]. As an example of this non-linear relationship, we can look to the results from AN^LO^ treatment administered to mice in our study. AN^LO^ mice received a dose of 24 mg/kg, which exceeds the current WHO single therapeutic dose (20 mg/kg) for treating human malaria, however, in our study this dose of pure drug had very little effect against mouse malaria parasites. This is most likely due to metabolic differences and rates of uptake of oral drug between mice and humans.

Single oral dosages of AN proven effective against human *P. falciparum* malaria range from 100–500 mg [41], which corresponds to 6–33 grams of WP assuming a 1.5% artemisinin content. Assuming an average tablet size of 1 gram, delivery of a comparable dose of WP seems plausible. However, our data suggests that WP requires a smaller overall amount of artemisinin since even the WP^LO^ effectively reduced malaria after just a single dose. Moreover, it is important to bear in mind that while WP^LO^ was the lowest concentration in our study, it does not necessarily represent a minimum effective dose.

**Figure 4:**
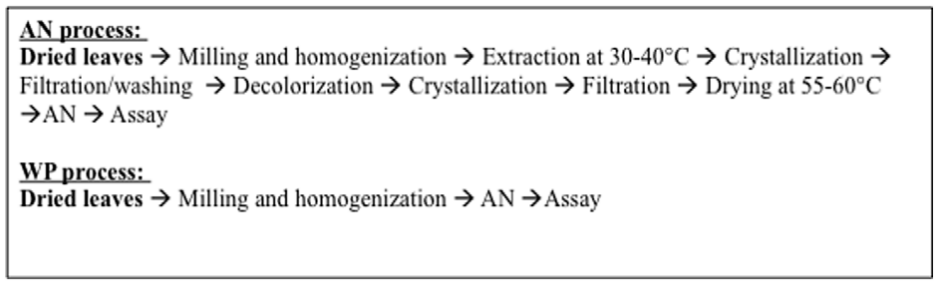
Comparison of unit operations required for production of extracted AN and production of WP. Extracted AN process is based on that described by de Vries et al. [45].

Notwithstanding challenges to be overcome and further research needed, our preliminary investigations hold great promise for easing the burden of high cost and limited availability currently confronting use of artemisinin-based semi-synthetic derivatives. While production costs for pharmaceuticals are not generally publically available, it is possible to estimate potential savings associated with using WP vs. AN by looking to the estimated efficiencies of processing AN from the plant. Kindermans et al [42] estimate the yield for extraction and purification of drug from *A. annua* to be 50–80% efficient. This is consistent with general understanding of downstream processing costs wherein product losses increase with the number of unit operations (unit ops) [43]. All things being equal (e.g. artemisinin content for a given cultivar), this would suggest a 20–50% savings realized by forgoing the extraction and purification steps, as would be the case for production of WP. An example of the steps involved in production of AN from whole plant is provided by de Vries et al., and compared with steps for producing WP (Figure 4). Current production of AN by extraction from *A. annua* has seven more unit ops than the one associated with direct use of the whole plant, and this net loss in efficiency translates to higher costs per product unit (Figure 4). Given the multiplicative principle of downstream processing, even if each processing step has relatively high efficiency, the overall efficiency will be less. For example, even if each of the seven additional steps for production of AN was 95% efficient, the overall efficiency would be merely 69.8%, which means that less than three quarters of the starting material is realized as drug. Such a loss translates to higher cost for the delivered drug. This simple analysis does not even consider the additional costs for reagents, labor, and energy that are required for processing the extract (Figure 4), which de Vries et al. [44] estimated may comprise up to 22.9% of the total manufacturing costs. This 22.9% represents additional cost savings for the use of WP because those inputs are no longer required.

And while our data are far from representing a clinical trial, they do provide preliminary indications that the WP therapy requires a far smaller dose than the corresponding amount of pure artemisinin. Indeed, our experiments indicate that a dose of WP has a five-fold increase in anti-malarial activity over that of the corresponding amount of AN. This increased activity per unit of plant mass not only affects the dosing regimen but also has profound economic impacts if the WP approach should prove useful on a large scale to treat human malaria.

Much work remains to determine feasibility and efficiency of bringing whole plant *A. annua* into the arsenal in the fight against malaria. Among the challenges to be faced are some botanical obstacles, not the least of which is that this plant readily outcrosses, making it difficult to maintain high artemisinin content in the plant using seed saving methods which are standard agricultural practice in the developing world. Moreover, to date all efforts at improving the *A. annua* crop have focused on plant breeding and agricultural methods to maximize artemisinin content and in so doing to increase efficiencies and drive down costs. The use of the whole plant as therapy may represent a paradigm shift in this regard, since it may well be the case that effectiveness of WP is not wholly dependent on artemisinin content. New plant breeding strategies would have to be considered to optimize plant performance and maximize efficacy. The potential for an inexpensive malaria therapy that by its very nature possesses great resilience to parasite resistance, makes this investment of effort well worthwhile.

## Conclusions

We have demonstrated that orally delivered WP *A. annua* is an effective means of killing malaria parasites in a mouse model. An edible WP *A. annua* treatment approach could significantly increase the number of patients treated and at significantly less cost. In fact, our results suggest that the WP treatment is a more efficient delivery mechanism than the purified drug, which is both costly and inefficient. Because AN has such broad potential therapeutic power against many infectious agents [3], our approach would dramatically reduce the cost of healthcare not only in developing countries, but also in more developed nations. Furthermore, use of *A. annua* could be implemented locally: a plan for plant cultivation, processing, and drug content validation was described in our earlier report [10]. This, in turn, could provide a broad socioeconomic stimulus for developing countries bearing the greatest burden of malaria transmission.

## Methods

### Plant material

*Artemisia annua* L. (SAM cultivar; voucher MASS 00317314) containing 1.48±0.06% AN (dry weight) as determined by GC-MS was used in this study. To obtain adequate amounts of leafy biomass, plants were grown in soil either in greenhouses, culture rooms, or growth chambers under continuous light to maintain vegetative growth. Plants were propagated by cuttings to insure that the outcrossing characteristics of *A. annua* did not result in genetic loss of AN content. When the plants reached about 0.5–1 m, they were harvested and dried in the light at 25°C for several days. Leaves were then stripped from stems and pulverized through a series of brass sieves ending with 600 µm meshed powder. All dry leafy biomass was pooled, homogenized and then assayed for AN.

AN was measured using GC-MS by extracting with pentane (∼6 mg/mL) and sonicating for 30 min. Extract was decanted into glass test tubes and dried under nitrogen gas, then stored at −20°C until analysis. Samples were resuspended in pentane and transferred to a 1 mL vial with a 100 µL glass insert. A 1 µL injection into the GC-MS [GC, Agilent 7890A; MS, Agilent 5975C; column, Agilent HP-5MS (30 m ×0.25 mm ×0.25 µm)] used the following oven program: ion source temperature 280°C; inlet 250°C; initial temperature of 125°C for 1 min, then ramp to 300°C at 5°C/min, for a total time of 36 min. Ultrapure helium was the carrier gas at 1 mL min^−1^. Identification was via NIST library and purchased AN standard (Sigma-Aldrich Chemical, St. Louis, MO).

### Parasite information

*Plasmodium chabaudi* ASS (MRA-429) was obtained through the Malaria Research and Reference Reagent Resource Center (MR4) as a part of the BEI Resources Repository, NIAID, NIH. Tubes of blood collected from infected mice, were removed from liquid nitrogen storage and left at room temperature for 30 minutes. A 100 µL aliquot of the parasite-infected blood was mixed with 500 µL Dulbecco's Phosphate Buffered Saline (DPBS). To activate parasite stocks, two C57BL/6 mice were injected intraperitoneally (i.p.) with 200 µL of the DPBS mixture. Percent parasitemia was determined in Giemsa-stained thin blood smears days 3–7 post-infection (p.i.). Seven days after infection, one mouse was euthanized and cardiac puncture was used to collect blood into lithium heparin tubes. Infected blood was volumetrically adjusted by dilution in DPBS to create a 200 µL aliquot of 10^5^ infected erythrocytes for infection into two additional mice for a second round of activation. The activated parasites were used for subsequent challenge and drug study. Rodent malaria models showed differences in the degree of susceptibility among different strains of mice, as well as age related differences. We used two mice to draw the standard parasitemia curve in the C57BL/6, 8–12 weeks male mice. The two mice were inoculated i.p with 10^5^ infected erythrocytes and parasitemia was determined in Giemsa-stained thin blood smears from day 1 to day 11 p.i. (Figure 1). *P. chabaudi* produces a self-limiting infection in laboratory mice, such that parasites began to appear in the blood smears on day 4 p.i., reaching peak of the parasitemia on day 8 p.i. From the standard parasitemia curve we chose to treat mice on day 6 p.i. when the parasites commenced the log phase of growth.

### Ethics Statement

This study was carried out in strict accordance with the recommendations in the Guide for the Care and Use of Laboratory Animals of the National Institutes of Health. The protocol was approved by the Institutional Animal Care and Use Committee (IACUC) of the University of Massachusetts (Protocol# 2011-0015). All efforts were made to minimize suffering of animals during experimental procedures.

#### Mouse feeding and drug delivery details

All mouse experiments used inbred male mice C57BL/6, 12 weeks of age. For each experimental replicate, an aliquot of 10^5^ infected erythrocytes was inoculated i.p. into each of 30 mice. For the first two replicates, mice were randomly divided into three groups of ten mice per group (AN^LO^, WP^LO^, and Control). For the third replicate, mice were randomly divided into five groups of six mice per group (AN^LO^, WP^LO^, AN^HI^, WP^HI^, and Control). Individual mice were identified by tail markings using permanent marker. Starting on day 3 p.i., percent parasitemia was determined in Giemsa-stained thin blood smears from a drop of peripheral blood obtained from the tail. Mice were observed twice daily for signs of disease stress. Food and water were introduced *ad libitum* for the first five days. On day five, food was withheld for 24 hours, but water was freely available.

Whole plant (WP) treatment consisted of dried *A. annua* plant powder ground and passed through a 0.3 mm sieve, mixed with water to a final volume of 0.5 mL. Two dosages were used: WP^LO^ and WP^HI^. WP^LO^ consisted of 0.5 mL treatment slurry contained 40 mg dry weight (DW) of plant powder, containing 600 µg artemisinin and corresponding to 24 mg AN/kg live body weight. WP^HI^ consisted of 0.75 mL treatment slurry contained 200 mg DW of plant powder, containing 3000 µg artemisinin and corresponding to 120 mg AN/kg live body weight.

Pure drug (AN) treatment consisted of artemisinin purchased from Sigma Aldrich freshly dissolved in DMSO and pulverized mouse chow. Two dosages were used, AN^LO^ and AN^HI^. AN^LO^ consisted of a slurry containing 600 µg AN dissolved in 60 uL DMSO mixed with water and 40 mg powdered mouse chow to final volume of 0.5 mL. AN^LO^ consisted of a slurry containing 3000 µg AN dissolved in 60 uL DMSO mixed with water and 200 mg powdered mouse chow to final volume of 0.75 mL.

Placebo control (CON) consisted of 60 µL DMSO, mixed with water and 40 mg powdered mouse chow to a final volume of 0.5 mL. Delivery of the appropriate 0.5 mL treatment/control was performed immediately after dose preparation by oral-gastric gavage into mice using a feeding needle (18G, curved, 2”, and 2.25 pall diameter). Food and water were introduced *ad libitum* after gavage. Percent parasitemia was determined every 24 hours in Giemsa-stained thin blood smears from days 3–6 p.i., then every six hours for 48 hours post gavage, and again on 24 hour intervals for days 9–11 p.i. All mice were euthanized on day 11 p.i. via asphyxiation in a CO_2_ chamber followed by cervical dislocation. The experiment was repeated three times.

### Statistical analysis

We fit linear mixed models to estimate and compare the average parasitemia for each treatment group at each measured time point. Including a random intercept for individual mice allowed us to adjust for repeated observations on the same mouse. The primary analysis compared CON, WP^LO^ and AN^LO^ treatment groups to assess statistically significant parasitemia differences in these groups at each time point (see Figure 2). For the primary analysis, data from all three replicates were used. A secondary dose-response analysis compared the CON, WP^LO^, WP^HI^, AN^LO^, and AN^HI^ treatment groups at all measured time points (see Figure 3). For the secondary analysis only data from the third replicate were used.

For each model, 10,000 Markov chain Monte Carlo (MCMC) samples were drawn from the posterior distributions of the average parasitemia levels for each treatment group at each time point. Then, 95% confidence interval endpoints for a particular parasitemia level were established at the 2.5^th^ and 97.5^th^ quantiles of the MCMC samples for that parameter. An estimated difference between two groups was declared “significant” if the 95% confidence interval for the difference did not cover zero. Analyses were conducted using the statistical software R v2.15 and the lmer package [45], Graphics were produced using the ggplot2 package [46], [47].

## References

[1] WHO (2010) World Malaria Report.

[2] de Ridder S, van der Kooy F, Verpoorte R (2008) *Artemisia annua* as a self-reliant treatment for malaria in developing countries. J Ethnopharmacol 120: 302–314. 18977424 10.1016/j.jep.2008.09.017

[3] Efferth T (2009) Artemisinin: a versatile weapon from traditional Chinese medicine. In: Ramawat KG, editor. Herbal drugs: ethnomedicine to modern medicine. Heidelberg: Springer Verlag. 179–194.

[4] Firestone GL, Sundar SN (2009) Minireview: modulation of hormone receptor signaling by dietary anticancer indoles. Mol Endocrinol 23: 1940–1947. 19837944 10.1210/me.2009-0149 PMC2796154

[5] Utzinger J, Xiao S, Keiser J, Chen M, Zheng J, et al (2001) Current progress in the development and use of artemether for chemoprophylaxis of major human schistosome parasites. Curr Med Chem 8: 1841–1860. 11772354 10.2174/0929867013371581

[6] Avery MA, Muraleedharan KM, Desai PV, Bandyopadhyaya AK, Furtado MM, et al (2003) Structure-activity relationships of the antimalarial agent artemisinin. 8. design, synthesis, and CoMFA studies toward the development of artemisinin-based drugs against leishmaniasis and malaria. J Med Chem 46: 4244–4258. 13678403 10.1021/jm030181q

[7] Sen R, Bandyopadhyay S, Dutta A, Mandal G, Ganguly S, et al (2007) Artemisinin triggers induction of cell-cycle arrest and apoptosis in *Leishmania donovani* promastigotes. J Med Microbiol 56: 1213–1218. 17761485 10.1099/jmm.0.47364-0

[8] Mishina YV, Krishna S, Haynes RK, Meade JC (2007) Artemisinins inhibit *Trypanosoma cruzi* and *Trypanosoma brucei* rhodesiense in vitro growth. Antimicrob Agents Chemother 51: 1852–1854. 17339374 10.1128/AAC.01544-06 PMC1855540

[9] Ferreira JF, Luthria DL, Sasaki T, Heyerick A (2010) Flavonoids from *Artemisia annua L*. as antioxidants and their potential synergism with artemisinin against malaria and cancer. Molecules 15: 3135–3170. 20657468 10.3390/molecules15053135 PMC6263261

[10] Weathers PJ, Arsenault PR, Covello PS, McMickle A, Teoh KH, et al (2011) Artemisinin production in *Artemisia annua*: studies in planta and results of a novel delivery method for treating malaria and other neglected diseases. Phytochem Rev 10: 173–183. 21643453 10.1007/s11101-010-9166-0 PMC3106422

[11] Barbacka K, Baer-Dubowska W (2011) Searching for artemisinin production improvement in plants and microorganisms. Curr Pharm Biotechnol 12: 1743–1751. 21902625 10.2174/138920111798376923

[12] Zhu C, Cook SP (2012) A concise synthesis of (+)-artemisinin. J Am Chem Soc 134: 13577–13579. 22866604 10.1021/ja3061479

[13] Kumar S, Gupta SK, Singh P, Bajpai MM, Gupta D, et al (2004) High yields of artemisinin by multi-harvest of *Artemisia annua* crops. Indust Crops Products 19: 77–90.

[14] Rodger WS, David ST (2012) Streamlined Process for the Conversion of Artemisinin to Artemether. Org Process Res Dev 16: 764–768.

[15] Graham IA, Besser K, Blumer S, Branigan CA, Czechowski T, et al (2010) The Genetic Map of *Artemisia annua L*. Identifies Loci Affecting Yield of the Antimalarial Drug Artemisinin. Science 327: 328–331. 20075252 10.1126/science.1182612

[16] Arsenault PR, Wobbe KK, Weathers PJ (2008) Recent advances in artemisinin production through heterologous expression. Curr Med Chem 15: 2886–2896. 18991643 10.2174/092986708786242813 PMC2821817

[17] Ro DK, Paradise EM, Ouellet M, Fisher KJ, Newman KL, et al (2006) Production of the antimalarial drug precursor artemisinic acid in engineered yeast. Nature 440: 940–943. 16612385 10.1038/nature04640

[18] Alin MH, Bjorkman A (1994) Concentration and time dependency of artemisinin efficacy against *Plasmodium falciparum in vitro* . Am J Trop Med Hyg 50: 771–776. 8024073 10.4269/ajtmh.1994.50.771

[19] Elford BC, Roberts MF, Phillipson JD, Wilson RJ (1987) Potentiation of the antimalarial activity of qinghaosu by methoxylated flavones. Trans R Soc Trop Med Hyg 81: 434–436. 3318019 10.1016/0035-9203(87)90161-1

[20] Liu KCSC, Yang SL, Roberts MF, Elford BC, Phillipson JD (1992) Antimalarial Activity of *Artemisia annua* Flavonoids from Whole Plants and Cell-Cultures. Plant Cell Rep 11: 637–640. 24213368 10.1007/BF00236389

[21] Cox FEG (1978) Concomitant Infections. In: Killick-Kendrick R, Peters W, editors. Rodent Malaria. London: Academic Press. 309–344.

[22] Ott KJ (1968) Influence of reticulocytosis on the course of infection of *Plasmodium chabaudi* and *Plasmodium berghei* . J Protozool 15: 365–369. 5703438 10.1111/j.1550-7408.1968.tb02138.x

[23] Carlton JM, Hayton K, Cravo PV, Walliker D (2001) Of mice and malaria mutants: unravelling the genetics of drug resistance using rodent malaria models. Trends Parasitol 17: 236–242. 11323308 10.1016/s1471-4922(01)01899-2

[24] WHO (2010) Guidelines for the Treatment of Malaria.

[25] Meshnick SR (2002) Artemisinin: mechanisms of action, resistance and toxicity. Internat J Parasitol 32: 1655–1660. 10.1016/s0020-7519(02)00194-7 12435450

[26] Meshnick SR, Thomas A, Ranz A, Xu CM, Pan HZ (1991) Artemisinin (qinghaosu): the role of intracellular hemin in its mechanism of antimalarial action. Mol Biochem Parasitol 49: 181–189. 1775162 10.1016/0166-6851(91)90062-b

[27] Svensson US, Ashton M (1999) Identification of the human cytochrome P450 enzymes involved in the in vitro metabolism of artemisinin. Br J Clin Pharmacol 48: 528–535. 10583023 10.1046/j.1365-2125.1999.00044.x PMC2014388

[28] Willcox M (2009) *Artemisia* species: From traditional medicines to modern antimalarials–and back again. J Altern Complement Med 15: 101–109. 19236169 10.1089/acm.2008.0327

[29] Rasoanaivo P, Wright CW, Willcox ML, Gilbert B (2011) Whole plant extracts versus single compounds for the treatment of malaria: synergy and positive interactions. Malaria J 10 Suppl 1S4. 10.1186/1475-2875-10-S1-S4 PMC3059462 21411015

[30] Bilia AR, Lazari D, Messori L, Taglioli V, Temperini C, et al (2002) Simple and rapid physico-chemical methods to examine action of antimalarial drugs with hemin: its application to *Artemisia annua* constituents. Life Sci 70: 769–778. 11833740 10.1016/s0024-3205(01)01447-3

[31] Lu J, Papp LV, Fang J, Rodriguez-Nieto S, Zhivotovsky B, et al (2006) Inhibition of mammalian thioredoxin reductase by some flavonoids: implications for myricetin and quercetin anticancer activity. Cancer Res 66: 4410–4418. 16618767 10.1158/0008-5472.CAN-05-3310

[32] Krnajski Z, Gilberger TW, Walter RD, Cowman AF, Muller S (2002) Thioredoxin reductase is essential for the survival of *Plasmodium falciparum* erythrocytic stages. J Biol Chem 277: 25970–25975. 12004069 10.1074/jbc.M203539200

[33] Bilia AR, Sannella AR, Vincieri FF, Messori L, Casini A, et al (2008) Antiplasmodial Effects of a few Selected Natural Flavonoids and their Modulation of Artemisinin Activity. Nat Product Comm 3: 1999–2002.

[34] Kraft C, Jenett-Siems K, Siems K, Jakupovic J, Mavi S, et al (2003) In vitro antiplasmodial evaluation of medicinal plants from Zimbabwe. Phytother Res 17: 123–128. 12601673 10.1002/ptr.1066

[35] Hsu E (2006) The history of qing hao in the Chinese materia medica. Trans R Soc Trop Med Hyg 100: 505–508. 16566952 10.1016/j.trstmh.2005.09.020

[36] van der Kooy F, Verpoorte R (2011) The Content of Artemisinin in the *Artemisia annua* Tea Infusion. Planta medica 77: 1754–1756. 21544776 10.1055/s-0030-1271065

[37] Weathers PJ, Towler MJ (2012) The flavonoids casticin and artemetin are poorly extracted and are unstable in an *Artemisia*. Planta Medica. 10.1055/s-0032-1314949 PMC3558976 22673829

[38] Duke J (2001) Handbook of phytochemical constituents of GRAS herbs and other economic plants. CRC Press LLC, Boca Raton, Fl: 70.

[39] Reagan-Shaw S, Nihal M, Ahmad N (2008) Dose translation from animal to human studies revisited. FASEB journal: official publication of the Federation of Am Soc Exp Biol 22: 659–661. 10.1096/fj.07-9574LSF 17942826

[40] Sharma V, McNeill JH (2009) To scale or not to scale: the principles of dose extrapolation. Br J Pharmacol 157: 907–921. 19508398 10.1111/j.1476-5381.2009.00267.x PMC2737649

[41] Gordi T, Huong DX, Hai TN, Nieu NT, Ashton M (2002) Artemisinin pharmacokinetics and efficacy in uncomplicated-malaria patients treated with two different dosage regimens. Antimicrob Agents Chemother 46: 1026–1031. 11897585 10.1128/AAC.46.4.1026-1031.2002 PMC127081

[42] Kindermans J-M, Pilloy J, Olliaro P, Gomes M (2007) Ensuring sustained ACT production and reliable artemisinin supply. Malaria J 6: 125. 10.1186/1475-2875-6-125 PMC2014776 17868471

[43] Atkinson B, Mavituna F (1991) Biochemical engineering and biotechnology handbook: Stockton Press.

[44] de Vries P, Chan N, de Goeje P (1999) Production and application of artemisinin in Vietnam: The Gioi Publishers.

[45] Team RDC (2012) R: A language and environment for statistical computing. In: R Foundation for Statistical Computing V, Austria.

[46] Douglas B, Martin M, Ben B (2011) lme4: Linear mixed-effects models using S4 classes. In: 0.999375-42 Rpv.

[47] Wickham H (2009) ggplot2: elegant graphics for data analysis. Springer New York.

